# An organ-on-chip device with integrated charge sensors and recording microelectrodes

**DOI:** 10.1038/s41598-023-34786-5

**Published:** 2023-05-18

**Authors:** Hande Aydogmus, Michel Hu, Lovro Ivancevic, Jean-Philippe Frimat, Arn M. J. M. van den Maagdenberg, Pasqualina M. Sarro, Massimo Mastrangeli

**Affiliations:** 1 ECTM, Department of Microelectronics, Delft University of Technology, Delft, 2628 CD The Netherlands; 2 Department of Human Genetics, Leiden University Medical Centre, 2333 ZC Leiden, The Netherlands; 3 Department of Neurology, Leiden University Medical Centre, 2333 ZC Leiden, The Netherlands

**Keywords:** Lab-on-a-chip, Biomedical engineering, Extracellular recording

## Abstract

Continuous monitoring of tissue microphysiology is a key enabling feature of the organ-on-chip (OoC) approach for in vitro drug screening and disease modeling. Integrated sensing units are particularly convenient for microenvironmental monitoring. However, sensitive in vitro and real-time measurements are challenging due to the inherently small size of OoC devices, the characteristics of commonly used materials, and external hardware setups required to support the sensing units. Here we propose a silicon-polymer hybrid OoC device that encompasses transparency and biocompatibility of polymers at the sensing area, and has the inherently superior electrical characteristics and ability to house active electronics of silicon. This multi-modal device includes two sensing units. The first unit consists of a floating-gate field-effect transistor (FG-FET), which is used to monitor changes in pH in the sensing area. The threshold voltage of the FG-FET is regulated by a capacitively-coupled gate and by the changes in charge concentration in close proximity to the extension of the floating gate, which functions as the sensing electrode. The second unit uses the extension of the FG as microelectrode, in order to monitor the action potential of electrically active cells. The layout of the chip and its packaging are compatible with multi-electrode array measurement setups, which are commonly used in electrophysiology labs. The multi-functional sensing is demonstrated by monitoring the growth of induced pluripotent stem cell-derived cortical neurons. Our multi-modal sensor is a milestone in combined monitoring of different, physiologically-relevant parameters on the same device for future OoC platforms.

## Introduction

Organ-on-chips (OoCs) are dynamic tissue culture devices that aim to mimic the microphysiological environments of organs in vitro. They have been employed to enhance the relevance of disease modeling and efficiency of drug development^1^. The integration of microfluidics into chips lead the field of cell-chemical formulation analysis^2^, which is crucial for, for instance, cytotoxicity monitoring^3^ and tumor diagnostics^4^. In recapitulating aspects of organ physiology on chip, however, several aspects should be taken into account, such as mechanical forces exerted on tissues, electrophysiological signaling among electrically active cell types, and monitoring biological cues in the extracellular matrix. These aspects of OoCs have the ability to improve the system’s reliability and physiological relevance^5^. In this respect, continuous and real-time monitoring of biological cues from cell cultures without terminal optical labeling techniques is crucial. Therefore, integration of multiple sensors into OoCs for real-time measurements is becoming the norm, especially for environmental cues, such as pH or oxygen levels^6^, whereby electro-chemical sensing is particularly convenient. The performance of the sensing units is thereby critical. In order to increase amplification of the output signal without the need of external circuitry, field-effect transistors (FETs) have been implemented as electro-chemical sensors to extract biochemically relevant information^7^. Depending on the coating of the transistor electrodes, selectivity towards specific analytes was demonstrated, as in the case of ion-sensitive FETs (ISFETs). ISFETs have been employed for more than 50 years to detect charge variations^8^. However, ISFETs have been usually in need of an external and bulky reference electrode, which can be hardly integrated into the inherently small-sized OoCs devices. Additionally, the reference electrode is usually based on Ag/AgCl and the charge variations in close proximity might ’turn-off’ the channel^9^. Moreover, FET-based sensors are usually fabricated on non-transparent substrates (e.g., silicon) which make them unsuitable to employ in OoC devices^10^.

**Figure 1:**
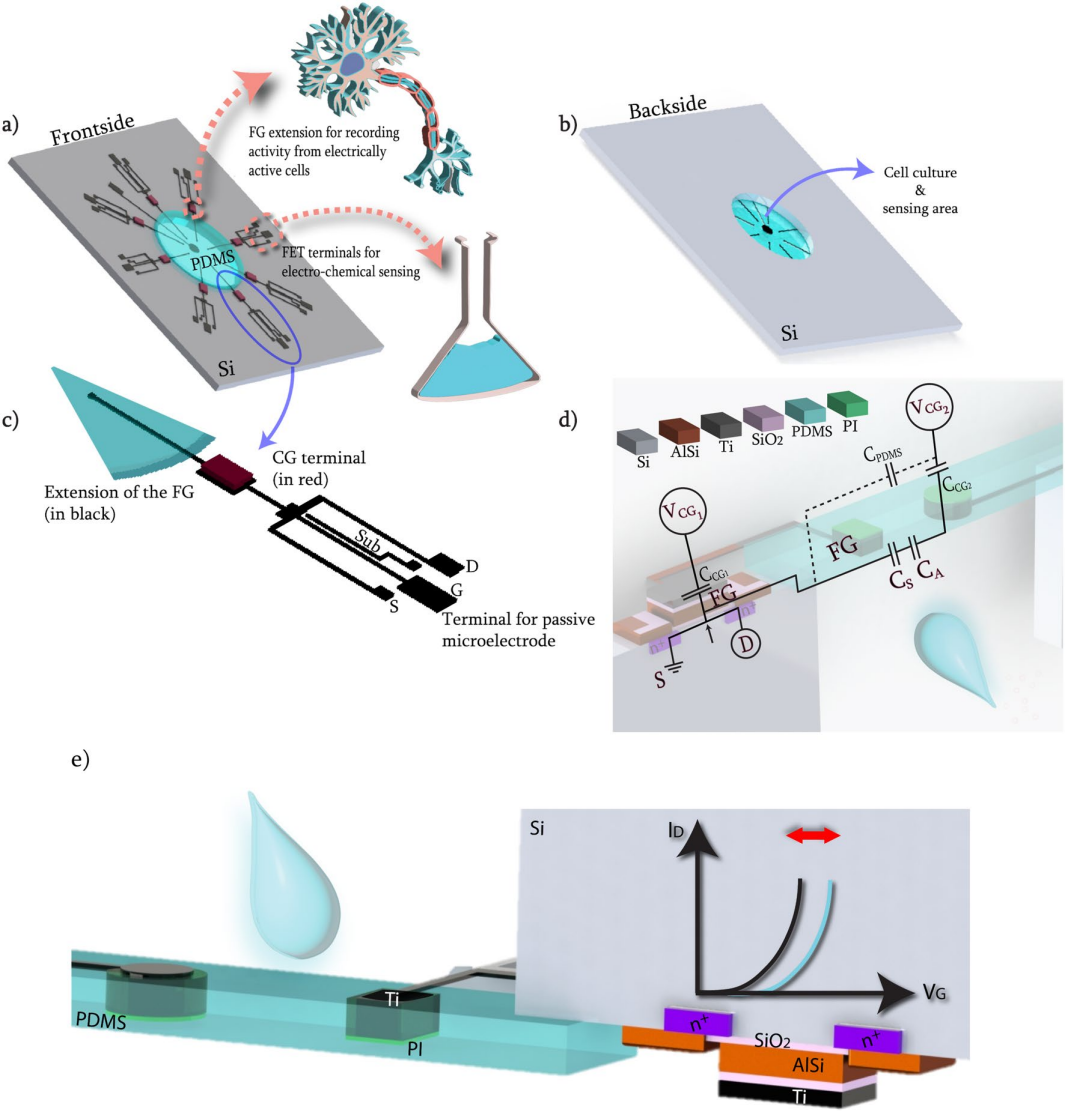
Schematic of the proposed OoC device with integrated multi-modal sensing. (**a**) Sketch of the frontside. Pink arrows indicate electrical connections for floating-gate FETs used to monitor pH changes, as well as extension of the floating-gates used as microelectrodes for recording activity from electrically active cells. (**b**) Sketch of the backside. Electrodes are fabricated on the frontside and the polymer membrane is released by etching silicon from the backside, leading to a well-like structure to house the analyte-under-test. (**c**) Sketch of one sensor with labeled terminals. (**d**) Schematic of the equivalent circuit of the device when used as FG-FET. Capacitances due to the electric double layer are shown as $C_S$ and $C_A$ at the sensing area. (**e**) Schematic of the working principle of the FG-FET. When there is a change in the net charge in close proximity to the FG extension, the working point of the sensor will change. This change can be detected by monitoring the drain current of the transistor.

As an alternative to these approaches, organic charge-modulated FETs (OCMFETs) were introduced. These are based on floating gates and are biocompatible and transparent, making them ideally suited for cell culturing applications on chip^9,11,12^. In this case, instead of using the external reference electrode, floating gates (FGs) were capacitively coupled to a control gate (CG) and used without applying voltage to the FG terminal to set the working point of the transistor. Detection of the analyte in close proximity of the extension of the FG electrode was realized by charge modulation with respect to the net charge, which in turn changed the working point of the transistor. Despite their distinctive signal-to-noise ratio, OCMFETs usually need application of high-voltage values to ’turn on’ the transistor. These values might disrupt cell’s physiology, depending on the organ model. Additionally, changes of the output signal due to charge modulation were reported on the levels of nA^7,12^, attesting to low sensitivity.

Here we present a silicon-polymer hybrid OoC device with integrated multi-modal electric (charge and field) sensing capabilities (Fig. 1). The device combines the superior electrical performance of silicon and biocompatibility, softness and transparency of polymers. The chip contains 8 FG-FET-based sensors, whose body and terminals lay on the peripheral silicon frame (Fig. 1a). The extension of the FGs goes over the central suspended polymer membrane, which makes up the tissue culture as well as sensing area, to track pH changes in real-time (Fig. 1b). Although the chip contains 8 FET-based sensors, for this study, only one of the sensors was used for tracking the pH changes, due to the current capability of the customised mobile measurement setup. The small size of the chip and particularly the size of the sensing area enables monitoring changes in small medium volume ($\sim 30\,\upmu \hbox {L}$). Additionally, depending on the terminal selection, extensions of the FG electrodes can be employed as microelectrodes, to monitor the activity of electrogenic cells such as neurons.

In the device presented here, FGs do not have terminals to apply voltage; rather, they are capacitively coupled to the environment and the control gates (CGs) (Fig. 1c). The first control gate ($CG_1$), which is on the silicon part, specifies the working point of the transistor, whereas the second CG ($CG_2$ in the schematic) completes the circuit and couples to the electrode-electrolyte interface (Fig. 1d). A net charge change in close proximity to the floating-gate extension (also referred to as the sensing electrode) induces a change in the threshold voltage of the transistor, and can be monitored as the change in drain current of the transistor; since the charge modulation on the FG induces a corresponding polarization of the dielectric of the gate (Fig. 1e). This transduction mechanism is detailed elsewhere^9,13^ and is also based on the polarization of the extension. Although the perfect polarization is not achievable experimentally, there is no current flowing through the FG extension, since the FG is only capacitively coupled and there is no leakage gate current, hence polarization is possible^14^.

**Figure 2:**
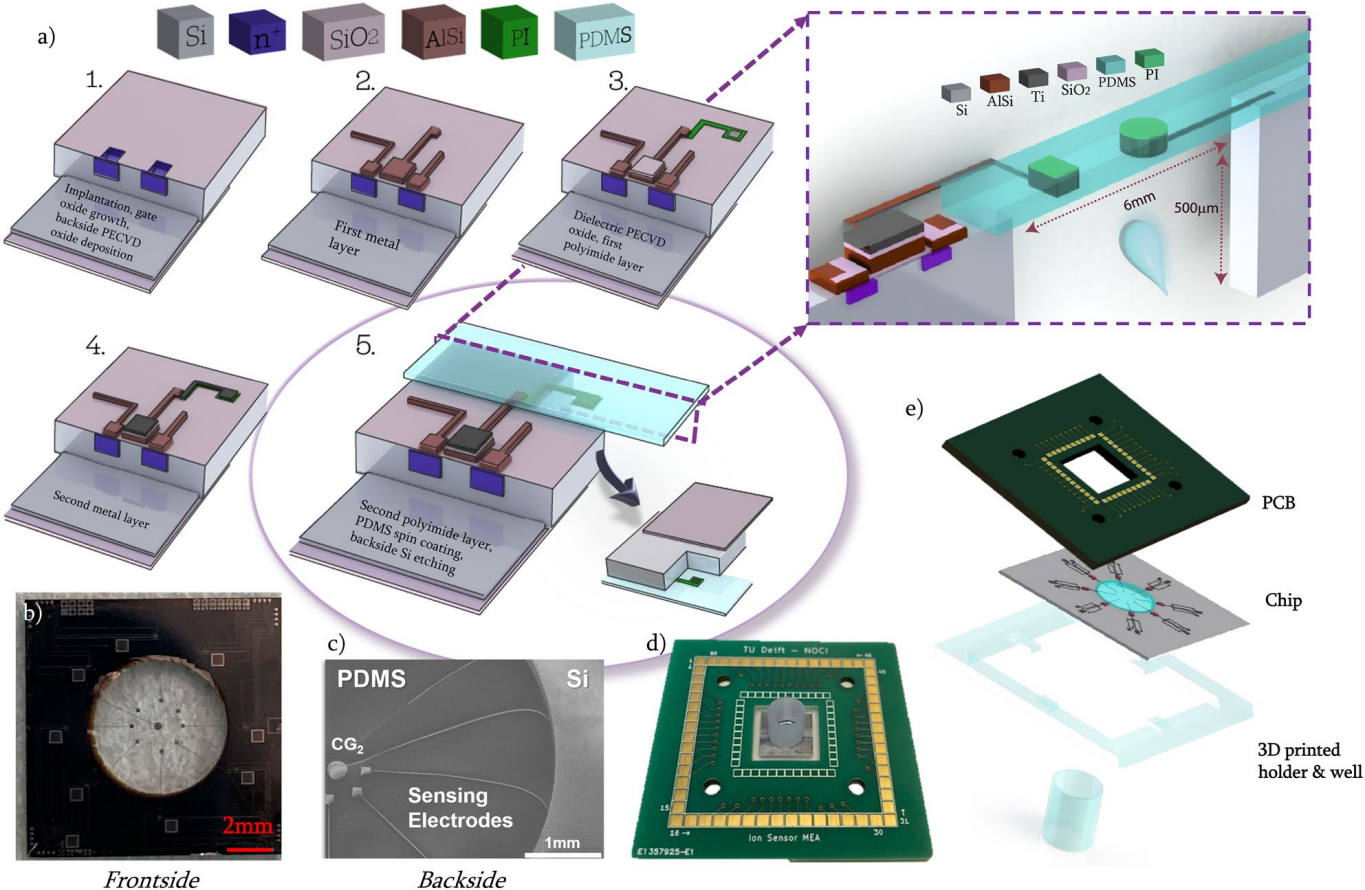
Fabrication and packaging of the OoC device. (**a**) Main steps in the fabrication of the sensor. Purple inset shows the dimensions of the final device, as well as the cross section. (**b**) Chip frontside after fabrication steps and dicing. (**c**) SEM image of the chip from the backside. (**d**) Assembled and ready-to-use device. (**e**) Schematic of the assembly of the chip, with 3D printed well and holder and the custom-designed PCB with central cut for optical imaging access.

Among human organs, the brain arguably has the most complex physiology, consisting of many different types of neurons and glial cells, interacting with each other in a complex manner. Monitoring the activity of electrically active cells, foremost neurons, can lead to the identification of dysfunctional neurobiological mechanisms underlying disease phenotypes. Researchers from interdisciplinary fields have been using electrodes for in vivo and in vitro recordings of action potentials^15^ in various setups, in particular micro-electrode arrays (MEAs). MEAs are usually implemented to record hundreds of neurons at the same time in a relatively high-throughput manner, which has been shown to be a robust functional readout to distinguish between healthy and diseased conditions^16^. Furthermore, MEAs have also been shown to be suitable for drug discovery and testing^17–20^. Various neuronal and glial cell types can be cultured on MEAs and form intricate neuronal networks. These neuronal networks can generate oscillations at various frequencies (from 0.05 Hz up to hundreds of Hz^21^). Different kinds of MEA devices exist including disposable^22^, 3D electrodes with optical stimulation^23^ and static MEAs integrated with impedance spectroscopy in a microfluidic device^24^ for optimizing signal extraction. Here, we use the extension of the FG electrodes as passive microelectrodes, to be able to use our chip as a MEA device, in addition to the already described charge sensing.

Reproducibility and standardization in OoC device fabrication are crucial for robust organ models. Therefore, BiCMOS-based wafer-level cleanroom fabrication methods were used to fabricate batches of nominally identical chips^25^. The fabrication flow is explained in detail in the “Methods” section, and the main steps are shown in Fig. 2a. The small size of the chips can be seen from the frontside (Fig. 2b) and the backside (Fig. 2c). To ease the handling of liquids at the sensing area, a 3D printed holder and a well were assembled on top of the chip (Fig. 2d). Additionally, custom-designed printed circuit boards (PCBs) compatible with commercially available microelectrode array read-out systems were designed to complete the packaging of the devices (Fig. 2e). This packaging makes the chips compliant with existing laboratory infrastructure and directly usable for action potential recordings of electrically-active cells without requiring additional technical knowledge of the end-user. In addition, a custom-made, compact and mobile electronic readout analyzer was developed to enhance the portability of the device, and to be able to use FG-FET sensors in relevant environments, e.g., incubators at $37\,\,^{\circ}C$ and $5\%$ $CO_2$.

## Results

### Modeling floating-gate FET as a pH sensor

One of the most important biochemical cues to monitor during cell culture is pH, since changes in pH can be caused by products of cell metabolism. Hence pH is an indicator of the viability of the cells, as well as of certain disease phenotypes^9,26^, as it is related to the medium acidification rate^27^.

The pH is a measure of concentration of hydrogen ions ($H^+$, i.e., protons), which is here modeled as the net charge. Various models to examine charging of surfaces have been developed in the last decades, here we use electrical double layer (EDL) and surface binding of charged species^28,29^. This model is based on both the site-binding theory and electric double layer capacitance^30^. In our model, relevant MOSFET parameters were extracted from dry measurements of the sensors with Advanced Design System (ADS), implemented into a custom Matlab code, and coupled to the equations for electrical double layer. The equations for the dynamics of the floating-gate and the electric double layer that is formed due to the electrolyte where then solved.

In theory, since there is no direct application of voltage to the FG, the trapped charge in the FG should remain constant. From the principle of conservation of charges, a charge relation can be established^7,9,12,14^. The FG voltage and, therefore, the change in drain current depends on three variables. The first variable is the surface charge ($\sigma _S$) due to the electrolyte-electrode interface coupled to $CG_2$, which impacts the surface potential at the sensing area ($\Psi _0$). This induces a potential $\Psi _S$ = $-\Psi _0$ through the metal underneath the oxide layer at the sensing area. The second variable is related to the trapped charge inside the FG ($Q_0$), and the last variable depends on $CG_1$(Eq. 1). Coupling of $CG_2$ through the PDMS membrane to the FG was also included in the equation, although $C_{P D M S}$ was calculated to be on the order of $\approx 10^{-16}F$, and thus negligible. These sources are summed for the final result for the FG voltage (Fig. 1d). Additionally, the second control-gate can be thought of as a pseudo-reference electrode, integrated by means of the planar cleanroom fabrication flow (Fig. 2a). As a result, all the elements in the circuit contribute to the FG voltage as the sum of capacitive couplings^31^:

1 $$\begin{aligned} V_{F G} = \frac{C_{{C G}_1} V_{{C G}_1}}{C_{t o t}} +\frac{Q_0}{C_{t o t}}+\frac{C_S\Psi _S}{C_{t o t}} +\frac{C_{P D M S} V_{{C G}_2}}{C_{t o t}} \end{aligned}$$

    The $CG_{2}$ (shown in Fig. 1d) modulates the potential at the sensing surface. The total capacitance $C_{t o t}$ includes the parasitic capacitance between the FG and the silicon body, $C_{CB}$, the capacitance from the PDMS membrane, $C_{P D M S}$, and the capacitance respectively of the sensing surface and the $CG_1$, $C_S$ and $C_{C G_1}$. The potential at the sensing surface $\Psi _S$ depends on $V_{FG}$, the potential at the EDL $\Psi _A$, and the corresponding surface charges $\sigma _S$ and $\sigma _A$ (See “Method” section for details). The drain current in the saturation region can be calculated from changes in threshold voltage:

2 $$\begin{aligned} I_D=\alpha (V_{F G}-V_{t h})^2 + \beta \end{aligned}$$

    Where $\beta$ is a fitting parameter which is included after experimental results (explained in the following section) and $\alpha$ is the constant associated to transistor characteristics:

3 $$\begin{aligned} \alpha = \frac{\mu _{e f f} w c_i}{2L}(1+\lambda (V_{D S} - V_{{D S}_{S A T}})) \end{aligned}$$

**Figure 3:**
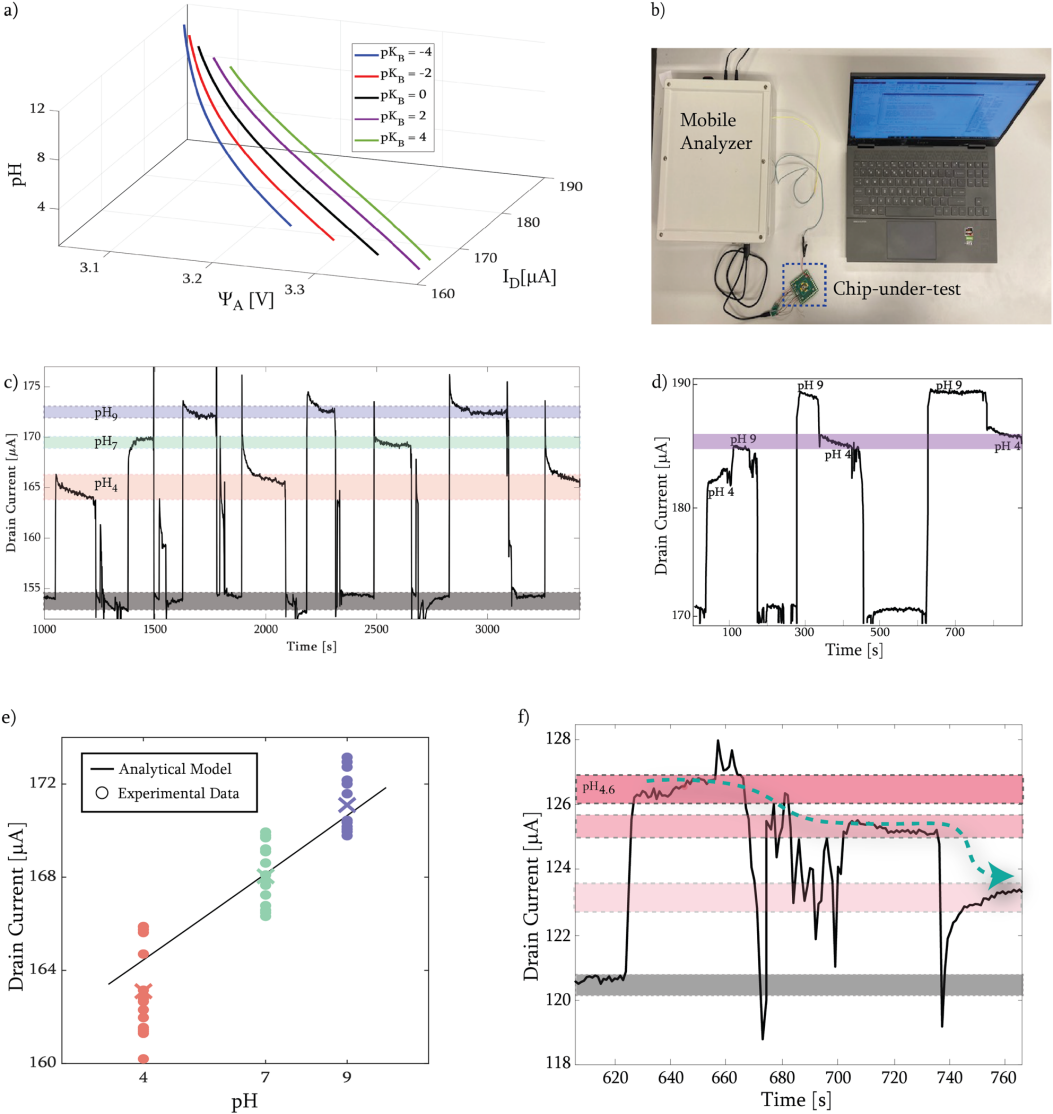
(**a**) Analytical model of the sensor with different dissociation constants. The 3D plot shows the change in potential at the EDL with respect to different pH and dissociation constants, and the resulting change in drain current. Experiments with pH calibration liquids: (**b**) Real-time pH sensing setup. The mobile measurement unit was connected to the chip-under-test, and resulting $I_D$ was monitored via Bluetooth connection on the computer. (**c**) Continuous monitoring of changes in pH (pink = pH 4, green = pH 7, blue = pH 9). Recording shows higher drain current shift when the initial value is higher compared to the other signals shown on the plot. (**d**) Saturation test on chip. pH 4 and pH 9 solutions of equal volumes were added sequentially without intermediate flushing. Resulting $I_D$ values are consistent with each other, as the resulting pH for all 3 events is the same. (**e**) Comparison between analytical solution and experimental data for the sensor represented at (**c**) . The ’X’ represents the mean value of the experimental dataset for each pH solution tested. (**f**) Sensitivity analysis with small decrements of pH. Arrow represents the increase in acidification due to sequential addition of pH 4 liquid. Downward spikes originate from the impact of the additional liquid at the sensing area.

    Binding of different ions and polar water molecules on the surface can be realized in different ways depending on different materials. Within the Matlab code, we characterized the surface potential at the sensing area for different surface dissociation constants and implemented it to the voltage of the FG and hence the drain current (Fig. 3a). The change in pH modulates the surface charge formation, and hence the change in potential at EDL and the sensing surface. These changes modulate the shift in threshold voltage and the drain current of the FET.

### Real-time pH measurements by using mobile measurement setup

The dry characterization of the FETs was described elsewhere^25^. For real-time monitoring of different pH values, the native TiO_2_ layer on the electrodes was used as selective layer and a compact and mobile measurement unit was developed (see “Methods” section). This unit provides a compact setup for real-time and continuous measurement. Portability and compactness are important since for OoCs the measurement environment can be an incubator in a cell biology laboratory, where external read-out equipment is not common, undesirable and, thus needs to be minimized (Fig. 3b). Before introducing the pH calibration liquids, the chip was flushed with deionized (DI) water and then dried. However, a thin layer of water molecules is expected to stay on the surface due to the surface binding. This can be considered as the initial state of the sensor (Fig. 3c, black band). Then, labeled buffer liquids with different pH values (4, 7 and 9, AVS TITRINORM, BDH Chemicals) were introduced to the sensing area, with the help of a micro-pipette ($\approx \,30\, \upmu$L). The order of introduction of liquids was random, to show the reproducibility of the sensor-under-test. Across consecutive measurements, the data showed high reversibility of the sensor, meaning that after removal of the solution and flushing with DI water, the sensor was back to its initial state within seconds, for the introduction of the next liquid (Fig. 3c). After each measurement, the tested liquid was removed from the sensing area with a micropipette, and the chip was flushed with DI water to reset the sensor reading to its baseline. Spikes caused by DI water handling between events can be observed in Fig. 3c. The average response time of the sensor was 5.48 s with a standard deviation of 1.3 s from 15 measurements (Supplementary Information, Fig. S1). While monitoring the measurements, the response time of the sensor was calculated from the instants when the readings were stable, before and after the update in pH of the solution (Supplementary Information, Fig. S2) and the mean and standard deviation values were shown in Supplementary Information, Fig. S3. For the preparation of the calibration dataset of the sensor with known pH values, we did not recirculate the buffer, to avoid any contamination. However, it might be useful to consider recirculation after constructing the calibration dataset of known pH values. Here, we chose an n-type device (nMOS), with $V_{C G} = 5V,$ $V_{D S} = 3V,$ $V_{S U B} = V_S = 0V$ and ${V_{CG_2}} = 3.3V.$ The drain current values were observed to decrease with increasing acidity, which is consistent with literature^7^, where for a p-type organic device the same trend in the opposite direction was reported. We assume that the higher shift from lower pH values originates from the polarization of the sensing oxide layer, sensing opposite charge of the actual formed layer. From the experimental data, we conclude that drain current values corresponding to different pH liquids (4, 7 and 9) could be measured with a few seconds of response time (Supplementary Information Fig. S1). From the measured data, a sensitivity value $\frac{\Delta I_{D}}{\Delta pH}$ was obtained as $1.5305\frac{\mu A}{pH}$ for the change between pH 7 and pH 9 and $1.3574\frac{\mu A}{pH}$ between pH 7 and pH 4.

To test the saturation of the device, without flushing the sensing area, the pH 9 buffer was added after the pH 4 buffer in equal volume. From the first event in Fig. 3d, we observed that when the pH 4 buffer was introduced, the increase of the current due to addition of the pH 9 buffer was lower, likely due to the fact that the surface of the sensing area was already mostly covered with H^+^ ions from the pH 4 solution. When the sensing area was cleaned and the pH 9 buffer was introduced first (second and third event), initial $I_D$ increased, and later decreased with the addition of pH 4 buffer, even though the shift in $I_D$ was lower when the sensor was at pristine condition prior to the introduction of the liquid. Resulting $I_D$ values were consistent with all 3 events for the same final pH (Fig. 3d, purple band). For pH sensing, we used native $TiO_2$ to serve as the selective layer^32^, and the surface dissociation constants $pK_A = 8$ and $pK_B = 4.5$^29,33^ to compare experimental results with the analytical model (Fig. 3e). We saw the same trend for drain current values both for analytical calculations and experiments (Supplementary Information Table 1). A fitting parameter ($\beta = 30$ $10^{-6}$A) was introduced in the model and summed to the drain current values to account for unknown parameters, i.e., Stern layer capacitance due to the electrical double layer (calculated to be $C_{Stern} = 1.078 \cdot 10^{-11}F$ with the assumptions based on^28^), the actual thickness of native $TiO_2$, and the trapped charge inside the FG.

Finally, using another n-MOS FG-FET on a separate chip, smaller changes of pH were recorded by sequential addition of 2 and 3 $\upmu \hbox {L}$ of pH 4 buffer (decreasing the initial pH of the liquid to 4.4 and 4.3, respectively) without flushing the sensing area (Fig. 3f). This test showed the sensitivity of the sensor to detect small (0.1) changes of pH in solution. Lower limits should be further investigated with a measurement setup with sub-nA current resolution, currently unavailable for our portable solution.

### Extracellular action potential recordings of neurons

**Figure 4:**
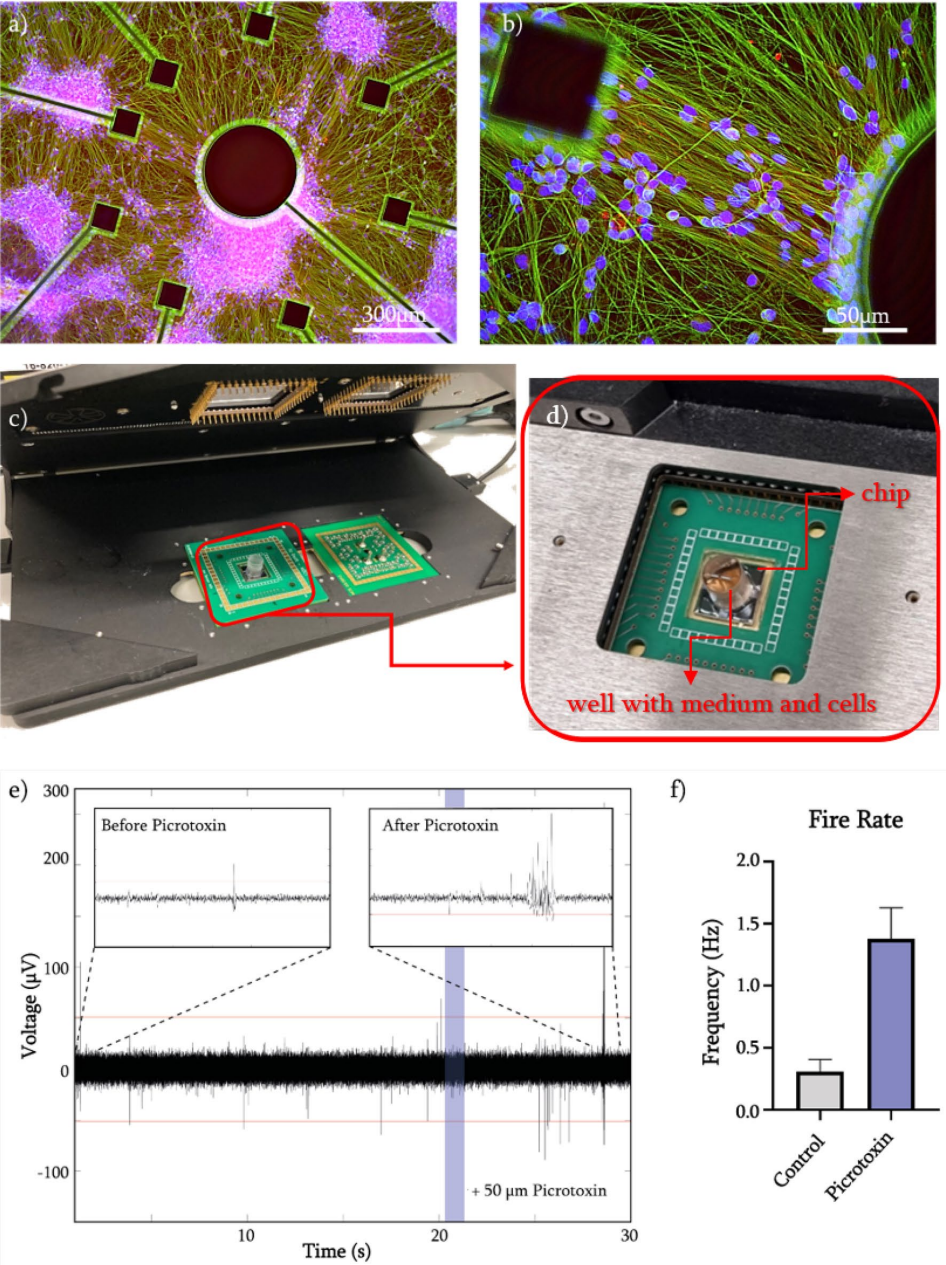
Biocompatibility and electrophysiological proof of concept experiments: (**a**, **b**) HiPSC-derived cortical neurons differentiated (7 days) and matured (14 days) on-chip. After staining, $\beta 3$ tubulin-positive neurons (green) and synaptic vesicles (red) are visible. (**c**) Compatibility with Multichannel Systems MEA recording setup of the PCB and the chip. (**d**) Inset showing the chip and the well with medium and cells. (**e**) Single-electrode trace signal from the MEA recordings. (**f**) The addition of Picrotoxin (50 $\upmu \hbox {M}$) caused an increase in fire rate.

As a proof-of-concept of the sensor, we use human induced pluripotent stem cell (hiPSC)-derived cortical neurons and show the sensor’s ability to record their action potential activity extracellularly. To this end, we introduced and used picrotoxin, a non-competitive $GABA_A$ antagonist, to induce burst-like behavior in our cell culture that can be detected using the MEA chip. Before recording extracellular spiking activity, the biocompatibility of the chip was tested. Neural progenitor cells (NPCs) were differentiated for 7 days and matured for 2 weeks on the chip before the cells were fixed and stained. Staining showed mature neuron marker ($\beta 3$ tubulin/green) as well as the production of synaptic vesicles (synaptophysin/red). The successful maturation of the hiPSC-derived cortical neurons confirmed the biocompatibility of the chips for live and non-invasive measurements (Fig. 4a,b).

For the recording of extracellular spike activity, we used the readout setup from Multichannel Systems (MCS), which is a commercially available and commonly used system for electrophysiology measurements in laboratories (Fig. 4c). The reason was to demonstrate compatibility with a standardized readout system and the ability of our chip to be used in any lab with this equipment, without the need of additional background knowledge of the end-user. We designed a PCB which was compatible with the MCS read-out setup for the 60-electrode MEA model. Inclusion of an opening at the center of the PCB allows end-users to have visibility of the cell culture due to the transparent PDMS membrane (Fig. 4d).

We recorded spontaneous electrophysiological signals of hiPSC-derived cortical neurons from one microelectrode, as a proof-of-concept. From the post-processing of the recorded signal, the average firing rate was 0.9661 Hz. Picrotoxin was added to the cell culture, to induce an epilepsy-like condition to the cell culture^34^ and to determine whether the chip could measure changes in electrophysiological signal of the neurons. We introduced the picrotoxin (50 $\upmu \hbox {M}$) 20 s after the recordings were started, as can be seen in Fig. 4e-blue line. Fig. 4f shows a representative channel that was recorded, and it was shown that the fire rate was increased on average (N = 3) from 0.31 ± 0.1 Hz before the picrotoxin was added to 1.38 ± 0.25 Hz after adding the drug. The resulting signal production from the hiPSC-derived neurons seen in Fig. 4f with a burst-like event (“after Picrotoxin” insert) is representative of exposure to picrotoxin.

## Discussion

We have shown the ability of the FG-FET-based sensor to measure local changes in pH and extracellular action potential recordings on-chip.

Our pH sensor revealed repeatability of the sensing while consecutively changing the liquid-under-test, without saturating the sensing electrodes under hour-long measurement periods. It is possible to pinpoint the pH value of the liquids due to their distinctive drain current fingerprints (Fig. 3d). Depending on the width and length of the channels of the FETs, different initial values of threshold voltage were obtained. The difference in the initial drain current values is caused by different gate dimensions and possible defects that might arise during fabrication. Calibration of each FET with pH buffer liquids would be necessary to obtain the fingerprint of the sensor, before testing liquids in physiologically relevant environments. For the experiments, after measuring the initial working point of the FET, values of pH changes were monitored. Concurrent measurements from multiple electrodes at once may induce cross-talk among electrodes and additional noise, deteriorating the signal-to-noise ratio. A guard-ring electrical insulation layer^35^ might be added during the device fabrication, to ensure removing possible cross-talk between close cellular environments.

Charge-modulated FG-FETs hold great promise, even though trapped charges in the FG are very small to manipulate without the use of a reference electrode (or pseudo electrode). If needed, applying voltage before experiments directly onto the FG would increase the trapped charges inside and hence would result in larger change in the threshold voltage. When our silicon-polymer hybrid sensor is compared with prior art^7,12^, we employ lower voltage values and obtain higher changes in current ($\upmu \hbox {A}$ rather than nA), which might increase the viability of cell cultures where long culturing periods are needed to examine various phenotypes-under-test. Additionally, the wafer-scale cleanroom fabrication process allows for a higher precision, reproducibility and yield of the sensors, and makes it possible to integrate multiple materials and more complex circuits in OoC devices, if desired. In our device, the sensing area and the electronic terminals are isolated from each other. Hence, without changing the intrinsic materials (e.g., gate oxide) of the transistors, selective coatings can be applied as a post-processing step^36^. This post-processing step allows different charge or affinity sensors to be realized.

As a proof-of-concept, here we used a low number of recording electrodes. However, due to cleanroom processing capabilities, the number of microelectrodes can be increased without compromising the small area of the chip (1 $\hbox {cm}^2$). The noise that can be coupled to the extracellular action potential recordings is mostly thermal^15^.

Activity of neuronal networks can be recorded at different frequencies, depending on the information that is desired to be examined. For the pH sensor, a sampling rate of 1 Hz was chosen. Although action potential events usually occur at higher frequencies, the same setup without modifications can also be used for electrophysiology. The spontaneous electrophysiological activity of the hiPSC-derived cortical neurons were successfully measured on chip and depending on the need, it is also possible to record local field potentials. Since the electrodes that have been employed for this study are relatively large compared to typical cell size (different sizes, up to 100 $\upmu \hbox {m}$) they might be good candidates for low-frequency local-field-potential recordings. Due to the $1/f^2$ noise, larger electrodes are more favourable^15^. Additionally, the potential differences on $CG_2$ and FG terminals can enhance the electric field and could be used to examine directional neurite growth^37^ and perhaps even determine the direction in which axons will grow. Galvanotropism studies have shown that is it possible to guide the growth of axons depending on the field strength. This could be a more natural approach as opposed to using topographical cues such as grooves to direct axons to mimic, for example, the spatial organization as seen in the cortex of brain-on-chip models^38–40^.

Although we have shown separately the working principle of the device for the two sensing modes, namely, tracking the drain current of the FETs for distinguishing liquids with different pH values, and recordings of extracellular action potential, in the future the measurements can be operated simultaneously on the same chip, to monitor both electrical activity and pH changes under physiologically-relevant conditions. The bimodality of the integrated sensor would reveal simultaneous measurements of change in charge concentration in the media and external action potentials from the cells, which might reveal relevant information about certain disease phenotypes and the viability of cells, without the need of transferring the media to analyze onto a different module. Real-time measurements of pH change from actual cell media over time are also part of future work. Finally, the chip can easily be integrated in OoC platforms, such as the translational organ-on-chip (TOP) platform^41^, due to its compactness and the usage of the mobile measurement setup. This would facilitate the study of multiple organ modules and monitoring of several biological cues with the microfluidic board which can be modeled for different gradients^42^ and programmed for fluid manipulation and biochemical assays^43^.

## Methods

### Fabrication of the sensor

The device is fabricated by means of a wafer-level CMOS-compatible process flow. A 4-inch, 525 $\upmu$m-thick, double-side polished p-type Si wafer was used for the fabrication of nMOS-based sensors using a standard BiCMOS process. After defining source and drain terminals by ion implantation, a 100 nm-thick gate oxide layer was thermally grown. A 6 $\upmu$m-thick oxide layer was deposited by plasma enhanced chemical vapor deposition (PECVD), and patterned on the wafer backside as etch hard mask (Fig. 2a,1). A 1 $\upmu \hbox {m}$-thick layer of $Al/1\%$Si was sputtered and patterned on the front side to implement electrical interconnects and floating gates (Fig. 2a,2). A 150 nm-thick PECVD oxide layer was used as dielectric between CG and FG electrodes. Polyimide (PI, Fujifilm LTC9305, Europe N.V, Belgium) was then spin-coated and patterned as insulating and mechanical transition layer to cover most of the extension of the floating gate onto the OoC sensing region (Fig. 2a,3). A sputtered 300 nm-thick layer of Ti was patterned to serve as CGs and FG extensions, as well as the microelectrodes used for action potential recordings (Fig. 2a,4). Polydimethylsiloxane (PDMS, Sylgrad 184, Dow Corning, Midland, MI, USA) was mixed with its curing agent in a 10:1 ratio, degassed, spin-coated and cured to serve as 20 $\upmu$m-thick membrane for the OoC cell culturing/charge sensing area. A 200 nm-thick AlSi was sputtered to serve as the protection layer on the PDMS membrane, which was later released by etching the Si substrate from the backside by deep reactive ion etching (Fig. 2a,5). The wafer was finally diced into 52 equal, square chips with a footprint of 1 $\hbox {cm}^2$. A 3D printed holder (MOIIN Tech Clear Resin, Hamburg, Germany) was then mounted and the chips were wire-bonded to custom-designed PCBs. Depending on the electrical contact terminal on the device, the sensors can be used as a FET-based charge sensor or microelectrode to capture action potential events from electrically-active cells. Lastly, 3D printed wells (diameter = 6 mm, height = 7 mm), (MOIIN Tech Clear Resin) were glued with PDMS to the sensing area to ease the handling of liquids (Fig. 2b).

### Analytical model for pH sensing

To analyze the FG-FET, the system of governing equations is solved in Matlab. These equations solve for potential at the floating-gate $V_{F G}$, potential at the sensing surface $\Psi _S$ and EDL $\Psi _{A}$, and corresponding surface charges $\sigma _S$ and $\sigma _{A}$^12^.

4 $$\begin{aligned} \Psi _S&= V_{F G} + \frac{A_S (\sigma _S -\sigma _A)}{C_{S G}} \end{aligned}$$

5 $$\begin{aligned} \Psi _A&= \Psi _S - \frac{A_S \sigma _S}{C_{S t e r n}} \end{aligned}$$

    Potential at the double layer affects the surface charge $\Psi _S$ and correlates the bulk potential of the solution ($V_{B u l k}$, assumed to be the same as the voltage applied from $CG_2$) to the surface. Additionally, $CG_2$ alters the potential at the EDL on top of the sensing surface:

6 $$\begin{aligned} \sigma _{A} =\frac{{C_{C G 2} (\Psi _A - V_{C G 2})}}{A_S} -B sinh \left( \frac{\Psi _A - V_{B u l k}}{2 k T}\right) \end{aligned}$$

    Capacitance due to $CG_2$ was calculated by assuming another Stern layer due to electrolyte-electrode interface and its native TiO_2_ layer. The surface charge due to hydroxyl groups at the oxide surface is related to the number of binding sites $N_s$ and surface dissociation constants $K_*$:

7 $$\begin{aligned} \sigma _S =\frac{-q N_s}{1 +\frac{H^+}{K_A}} exp\left( -q \frac{(\Psi _S - V_{B u l k})}{k T}\right) + \frac{q N_s}{1 +\frac{K_B}{H^+}} exp\left( q \frac{(\Psi _S - V_{B u l k})}{k T}\right) \end{aligned}$$

    The change in the threshold voltage appears from the net charge at the sensing surface ($Q_S$) and initial threshold voltage, which is obtained from the CG:

8 $$\begin{aligned} V_{t h} = \frac{V_{{t h},_0} C_{C G_1}}{C_{t o t}} +\frac{Q_0}{C_{t o t}} + \frac{ Q_S}{C_{t o t}} \end{aligned}$$

    These system of equations were used to calculate the behavior of the FG-FET-based sensor for different pH and dissociation constant values.

### Mobile measurement setup

For pH sensing, a mobile measurement setup was developed (Fig. 3c). The device includes a sensing board to bias the floating-gate FET and convert output current signal to voltage (adjusted via switchable resistors to sense even in the nA range). Furthermore, an 18-bit analog-to-digital converter (ADC) was employed to convert the voltage values to a digital code, and transmission of the signal was implemented via Bluetooth Low Energy. The output signal was monitored by a MATLAB script as well as an Android device.

### Human induced pluripotent stem cell (hiPSC)-derived cortical neuronal cultures

The hiPSC line LUMC0114iCTRL01 (hPSCreg number LUMCi003-A)^44^ was used to derive neural progenitor cells (NPCs) using the STEMdiff SMADi Neural Induction kit (05835, StemCell Technologies). The chip was plasma treated (50 W, 50 KHz, 45 s with CUTE Plasma System from Femto Science, Selangor, Malaysia) prior to coating with Poly-L-Ornithine (P3655,Sigma Aldrich) with a concentration of 100 $\upmu$g/mL and incubated at room temperature (RT) overnight. The next day the chip was incubated at $4\,\,^{\circ }\hbox {C}$ for 30 min before a laminin coating ($200\,\frac{\upmu \hbox {g}}{\hbox {mL}}$) was applied. After which the chip was incubated at $37\,\,^{\circ }\hbox{C}$ for 2 h. NPCs were seeded at a concentration of 100.000 cells $\cdot \hbox {cm}^{-2}$ on the chip and were subsequently differentiated into cortical neurons for 7 days using the STEMdiff Midbrain Neuron Differentiation Kit (100-0038, StemCell Technologies). Lastly, the hiPSC-derived cortical neurons were matured and maintained in BrainPhys hiPSC Neuron kit media (05795, StemCell Technologies) for the remainder of the experiment. All media were supplemented with $1 \%$ penicillin/streptovidin. For the drug experiments, to block inhibition, picrotoxin (P1675-1G, Sigma Aldrich) was prepared at a concentration of 50 $\upmu \hbox {M}$ in BrainPhys medium and was added 20 seconds after the recordings were started.

### Multi-electrode array (MEA) recordings

The MEA2100 system (Multi Channel Systems, Reutlingen, Germany) was used to record the electrophysiological activity of the neurons on chip with the heating stage set at $37\,\,^{\circ }\hbox{C}$. Several recordings of 1 min at 10 kHz were taken at days in vitro (DIV) 21 (i.e., 7 days differentiation and 14 days matured). The data was collected with a Butterworth High-Pass filter with a cutoff frequency of 200 Hz. Raw recording files (.msrd) were obtained from the MEA2100 system and were converted into HDF5 files using MCS software. We used MEA-ToolBox^45^ to analyze the MEA data in MATLAB 2018b (Mathworks, Massachusetts, USA). MEA-ToolBox was set at threshold $5 \cdot RMS$ for spike detection and the mean was calculated from 3 recordings.

### Immunohistochemistry

The hiPSC-derived cortical neurons were fixed at DIV 21 in $4 \%$ paraformaldehyde for 20 min at room temperature (RT) and permeabilized with $1 \%$ Triton X-100 (Sigma-Aldrich, St. Louis, MI, USA) in phosphate-buffered saline (PBS, Sigma-Aldrich) for 20 min. After the permeabilization step, the cells were washed three times in PBS for 5 min each at RT. Cells were then incubated with $1 \%$ bovine serum albumin (BSA; no 9048468, SigmaAldrich) for 30 min to block non-specific binding and again washed in PBS three times for 5 min each at RT. The primary antibodies used were $\beta 3$ tubulin (rabbit polyclonal PA5-86069, Thermofisher, Waltham, MA, USA), synaptophysin (mouse monoclonal ab8049, Abcam, Cambridge, UK). These primary antibodies were diluted 1:200 in $1 \%$ BSA in dPBS (does not contain magnesium and calcium) and incubated for 1 h at RT. The secondary antibodies were Texas red (Goat anti-mouse, ab7066, Abcam) and Alexa Fluor®488 nm (Goat anti-rabbit, ab150077, Abcam) which were diluted 1 : 500 in $1 \%$ BSA in dPBS. These secondary antibodies were incubated for 1 h at RT in dark before washed with PBS three times for 5 min each. Cell nuclei were stained using NucBlue (Life Technologies, Carlsbad, CA, USA) for 20 min. Fluorescent images were taken using a Keyence BZ-810 microscope system (Osaka, Japan).

## Supplementary Information

Supplementary Information.

## Data Availability

The datasets generated during the current study are available from the corresponding author. Analysed datasets are included in this published article.

## References

[1] Marx U (2016). Biology-inspired microphysiological system approaches to solve the prediction dilemma of substance testing. ALTEX.

[2] Preetam S (2022). Emergence of microfluidics for next generation biomedical devices. Biosens. Bioelectron..

[3] Bendre A (2022). Recent developments in microfluidic technology for synthesis and toxicity-efficiency studies of biomedical nanomaterials. Mater. Today Adv..

[4] Bhat MP (2022). Recent advances in microfluidic platform for physical and immunological detection and capture of circulating tumor cells. Biosensors.

[5] Mastrangeli M, van den Eijnden-van Raaij J (2021). Organs-on-chip: The way forward. Stem Cell Rep..

[6] Zhang YS (2017). Multisensor-integrated organs-on-chips platform for automated and continual in situ monitoring of organoid behaviors. Proc. Natl. Acad. Sci..

[7] Spanu A (2017). A reference-less ph sensor based on an organic field effect transistor with tunable sensitivity. Org. Electron..

[8] Bousse L, De Rooij NF, Bergveld P (1983). Operation of chemically sensitive field-effect sensors as a function of the insulator-electrolyte interface. IEEE Trans. Electron Devices.

[9] Demelas M (2013). Charge sensing by organic charge-modulated field effect transistors: Application to the detection of bio-related effects. J. Mater. Chem. B.

[10] Chen B, Parashar A, Pandey S (2011). Folded floating-gate cmos biosensor for the detection of charged biochemical molecules. IEEE Sens. J..

[11] Barbaro M, Bonfiglio A, Raffo L (2005). A charge-modulated fet for detection of biomolecular processes: Conception, modeling, and simulation. IEEE Trans. Electron Devices.

[12] Spanu A (2015). An organic transistor-based system for reference-less electrophysiological monitoring of excitable cells. Sci. Rep..

[13] Thomas MS, White SP, Dorfman KD, Frisbie CD (2018). Interfacial charge contributions to chemical sensing by electrolyte-gated transistors with floating gates. J. Phys. Chem. Lett..

[14] Spanu, A. *Organic tRansistor Devices for In Vitro Electrophysiological Applications* (Springer, 2016).

[15] Obien MEJ, Deligkaris K, Bullmann T, Bakkum DJ, Frey U (2015). Revealing neuronal function through microelectrode array recordings. Front. Neurosci..

[16] Mossink B (2021). Human neuronal networks on micro-electrode arrays are a highly robust tool to study disease-specific genotype-phenotype correlations in vitro. Stem Cell Rep..

[17] Cao Z (2012). Clustered burst firing in fmr1 premutation hippocampal neurons: Amelioration with allopregnanolone. Hum. Mol. Genet..

[18] Bateup HS (2013). Excitatory/inhibitory synaptic imbalance leads to hippocampal hyperexcitability in mouse models of tuberous sclerosis. Neuron.

[19] Wainger BJ (2014). Intrinsic membrane hyperexcitability of amyotrophic lateral sclerosis patient-derived motor neurons. Cell Rep..

[20] Bradley JA, Luithardt HH, Metea MR, Strock CJ (2018). In vitro screening for seizure liability using microelectrode array technology. Toxicol. Sci..

[21] Buzsaki G, Draguhn A (2004). Neuronal oscillations in cortical networks. Science.

[22] Charkhkar H (2016). Novel disposable microelectrode array for cultured neuronal network recording exhibiting equivalent performance to commercially available arrays. Sens. Actuators B Chem..

[23] Shin H (2021). 3d high-density microelectrode array with optical stimulation and drug delivery for investigating neural circuit dynamics. Nat. Commun..

[24] Zitzmann FD (2017). A novel microfluidic microelectrode chip for a significantly enhanced monitoring of npy-receptor activation in live mode. Lab Chip.

[25] Aydogmus, H. *et al*. Fet-based integrated charge sensor for organ-on-chip applications, in *2020 IEEE Sensors*, 1–4 (IEEE, 2020).

[26] Odijk M (2015). Microfabricated solid-state ion-selective electrode probe for measuring potassium in the living rodent brain: Compatibility with dc-eeg recordings to study spreading depression. Sens. Actuators B Chem..

[27] Müller B (2021). Measurement of respiration and acidification rates of mammalian cells in thermoplastic microfluidic devices. Sens. Actuators B Chem..

[28] Kaisti M (2015). An ion-sensitive floating gate fet model: Operating principles and electrofluidic gating. IEEE Trans. Electron Devices.

[29] Van Hal R, Eijkel J, Bergveld P (1996). A general model to describe the electrostatic potential at electrolyte oxide interfaces. Adv. Colloid Interface Sci..

[30] Kaisti M, Zhang Q, Levon K (2017). Compact model and design considerations of an ion-sensitive floating gate fet. Sens. Actuators B Chem..

[31] Shibata T, Ohmi T (1992). A functional mos transistor featuring gate-level weighted sum and threshold operations. IEEE Trans. Electron Devices.

[32] Manjakkal L, Szwagierczak D, Dahiya R (2020). Metal oxides based electrochemical ph sensors: Current progress and future perspectives. Progress Mater. Sci..

[33] Lin S-H, Chiou C-H, Chang C-K, Juang R-S (2011). Photocatalytic degradation of phenol on different phases of TiO_2_ particles in aqueous suspensions under uv irradiation. J. Environ. Manag..

[34] Rho JM, Donevan SD, Rogawski MA (1996). Direct activation of gabaa receptors by barbiturates in cultured rat hippocampal neurons. J. Physiol..

[35] Neamen, D. A. *Semiconductor Physics and Devices: Basic Principles* (McGraw-hill, 2003).

[36] Avdogmus, H. *et al*. Dual-gate fet-based charge sensor enhanced by in-situ electrode decoration in a mems organs-on-chip platform, in *2021 21st International Conference on Solid-State Sensors, Actuators and Microsystems (Transducers)*, 180–183 (IEEE, 2021).

[37] Deumens R (2004). Alignment of glial cells stimulates directional neurite growth of cns neurons in vitro. Neuroscience.

[38] Gokoffski KK, Jia X, Shvarts D, Xia G, Zhao M (2019). Physiologic electrical fields direct retinal ganglion cell axon growth in vitro. Investig. Ophthalmol. Vis. Sci..

[39] Yamashita M (2015). Weak electric fields serve as guidance cues that direct retinal ganglion cell axons in vitro. Biochem. Biophys. Rep..

[40] McCaig, C. D., Rajnicek, A. M., Song, B. & Zhao, M. Controlling cell behavior electrically: Current views and future potential. *Physiol. Rev.* (2005). 10.1152/physrev.00020.2004 15987799

[41] Vollertsen AR (2021). Facilitating implementation of organs-on-chips by open platform technology. Biomicrofluidics.

[42] Micheli S, Mocellin P, Sorgato M, Bova L, Cimetta E (2022). Modeling-based design specifications for microfluidic gradients generators for biomedical applications. Biochem. Eng. J..

[43] Zhang, D., Li, W., Shang, Y. & Shang, L. Programmable microfluidic manipulations for biomedical applications. *Eng. Regen.* (2022).

[44] Buijsen RA (2018). Generation of 3 spinocerebellar ataxia type 1 (sca1) patient-derived induced pluripotent stem cell lines lumci002-a, b, and c and 2 unaffected sibling control induced pluripotent stem cell lines lumci003-a and b. Stem Cell Res..

[45] Hu, M. *et al*. Mea-toolbox: An open source toolbox for standardized analysis of multi-electrode array data. *Neuroinformatics* 1–16 (2022). 10.1007/s12021-022-09591-6 PMC9588481 35680724

