# Supplementary material for: An organ-on-chip device with integrated charge sensors and recording microelectrodes
Source: Sci Rep. 2023 May 18;13:8062. doi: 10.1038/s41598-023-34786-5 (PMC10195821; doi:10.1038/s41598-023-34786-5)
Supplement: Supplementary file 1 — Supplementary Information. [file 41598_2023_34786_MOESM1_ESM.pdf]

An Organ-on-Chip Device with Integrated Charge Sensors and Recording Microelectrodes

Hande Aydogmus[1,*], Michel Hu[2,3], Lovro Ivancevic[1], Jean-Philippe Frimat[2,3], Arn M.J.M. van den Maagdenberg[2,3], Pasqualina M. Sarro[1], and Massimo Mastrangeli[1]

Supplementary Material

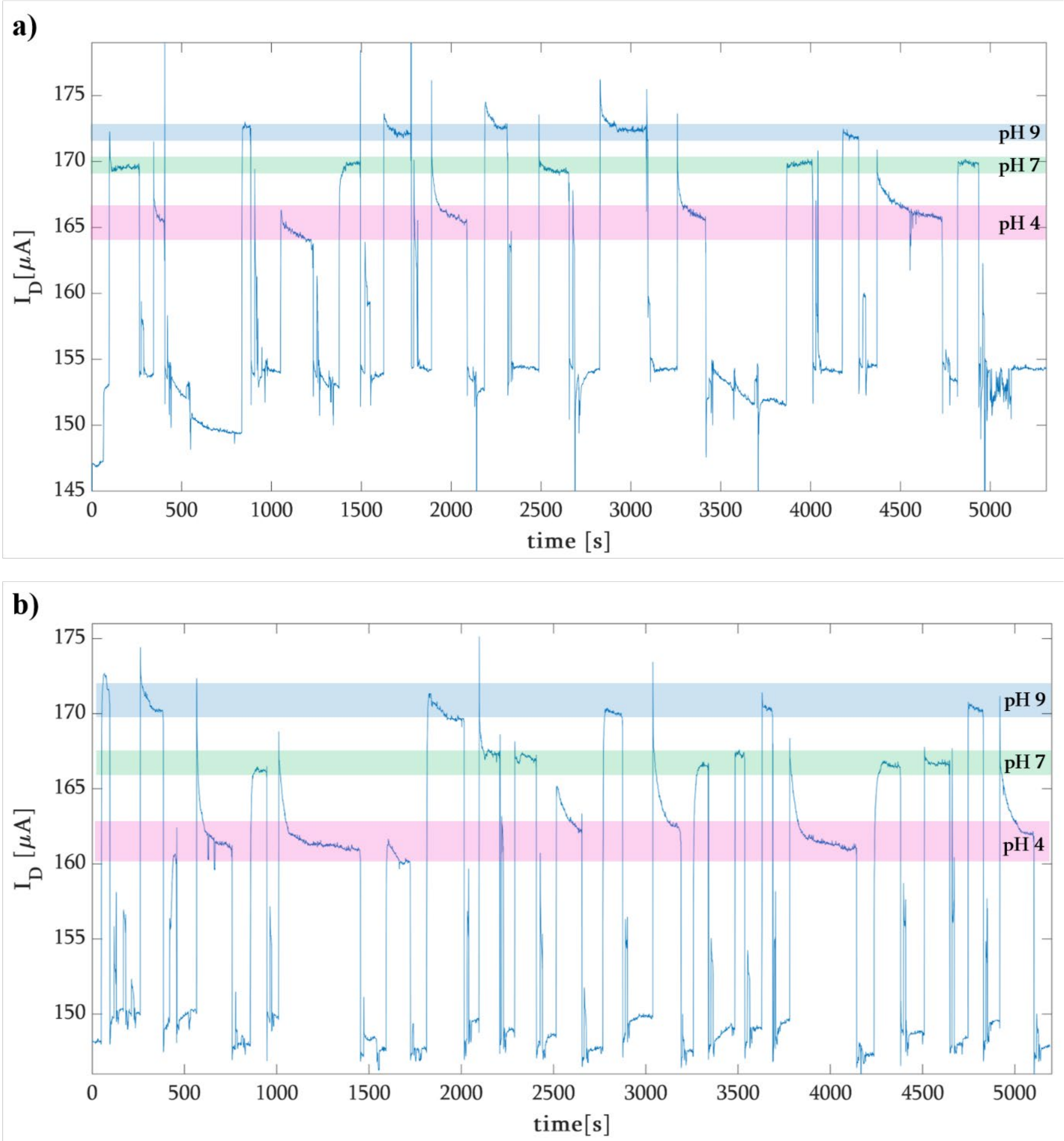

Figure S1: Raw data of the I_D recordings, 2 weeks apart in a) and b), respectively. pH 4, 7 and 9 buffer liquids induce shift in the output current I_D of the same NMOS-based sensor.

a)

pH	I_D_init[μA]	I_D_fin[μA]
7	153.065	169.211
4	153.781	165.625
9	149.365	172.73
4	154	164.7
7	152.979	169.793
9	153.742	172.167
4	154.191	165.749
9	152.741	172.949
7	154.42	169.097
9	154.143	173.15
4	154.286	165.864
7	151.635	169.697
9	154.086	172.005
4	154.639	165.883
7	153.161	169.936

b)

9	148.144	171.586
9	150.138	170.174
4	150.328	161.296
7	148.030	166.321
4	149.918	161.381
4	147.830	160.199
9	147.677	169.783
7	149.623	167.659
7	148.926	167.247
4	148.602	162.306
9	147.801	169.955
4	149.880	162.659
7	147.582	166.579
7	149.165	167.237
9	149.155	170.355
4	149.403	161.544
7	147.315	166.474
7	148.907	166.779
9	148.373	170.441
4	149.546	161.973

Table S1: Corresponding initial and final I_D values of the measurements shown in Figure S1.

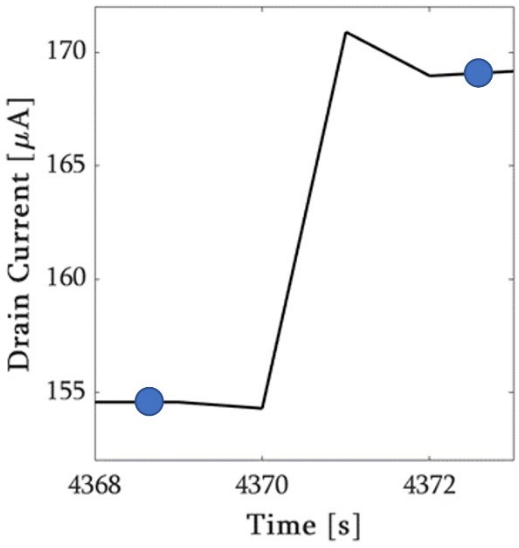

Figure S2: Drain current vs. time of an event from Figure S1. The average response time of the sensor is 5.48s with a standard deviation of 1.3s from 15 events. While monitoring the measurements, the response time of the sensor was calculated from the instants when the readings, before and after the update in pH of the solution, were stable.

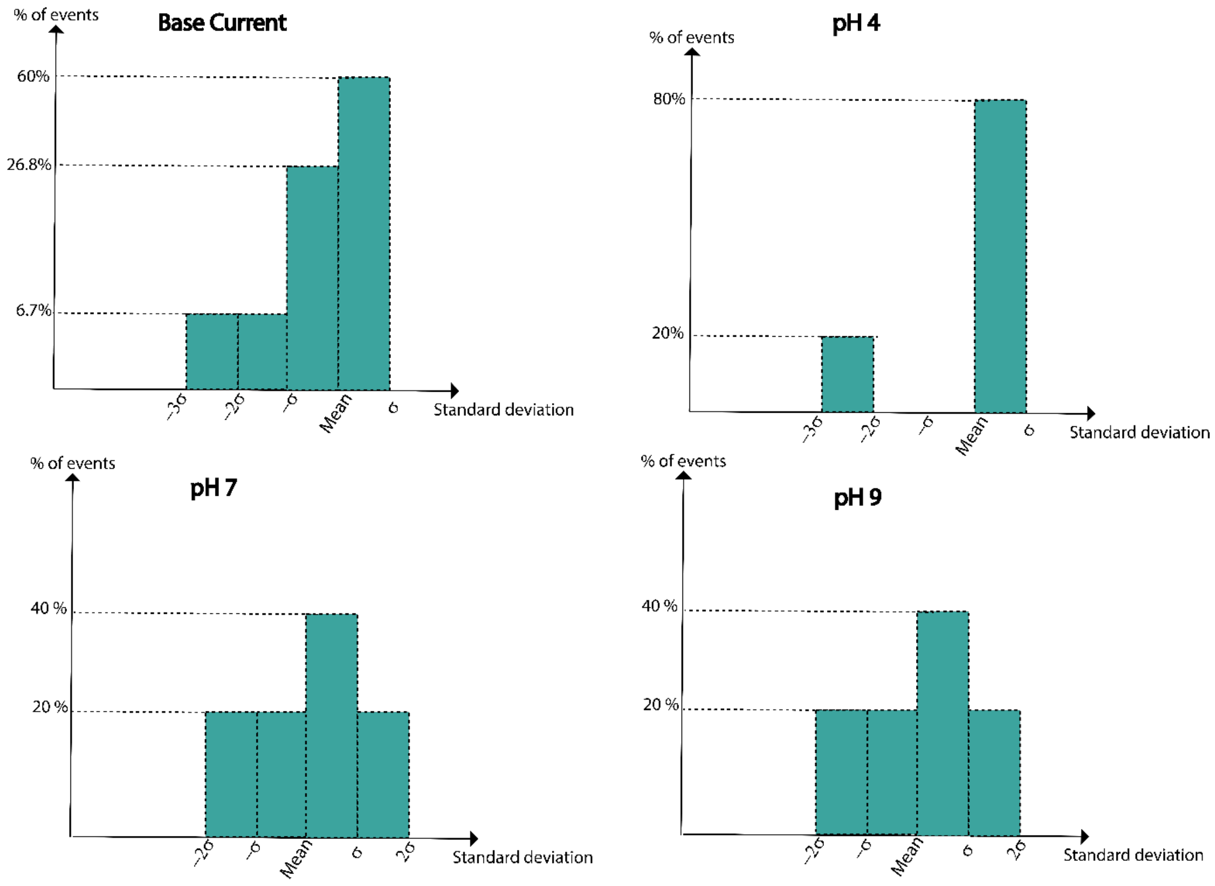

Figure S3: Mean and standard deviation values of the base drain current of the sensor and measurements with buffer liquids with different pH values. The values are shown in Fig 3.c and S1.
